# Global COVID-19 case fatality rates influenced by inequalities in human development and vaccination rates

**DOI:** 10.1007/s44155-022-00022-0

**Published:** 2022-11-01

**Authors:** Kaamel Nuhu, Kamal Humagain, Genevieve Alorbi, Sabena Thomas, Alexis Blavos, Vierne Placide

**Affiliations:** 1 Health Department, State University of New York at Cortland, Cortland, USA; 2 Geology Department, State University of New York at Potsdam, Potsdam, USA; 3 Economics Department, State University of New York at Oswego, Oswego, USA; 4 College of Nursing and Public Health, Adelphi University, Garden City, USA

**Keywords:** COVID-19, Case fatality rates, Population median age, Inequalities in human development, Healthcare capacity, Pandemic mitigation

## Abstract

**Aim:**

COVID-19 has exerted distress on virtually every aspect of human life with disproportionate mortality burdens on older individuals and those with underlying medical conditions. Variations in COVID-19 incidence and case fatality rates (CFRs) across countries have incited a growing research interest regarding the effect of social factors on COVID-19 case-loads and fatality rates. We investigated the effect of population median age, inequalities in human development, healthcare capacity, and pandemic mitigation indicators on country-specific COVID-19 CFRs across countries and regions.

**Subject and methods:**

Using population secondary data from multiple sources, we conducted a cross-sectional study and used regional analysis to compare regional differences in COVID-19 CFRs as influenced by the selected indicators.

**Results:**

The analysis revealed wide variations in COVID-19 CFRs and the selected indicators across countries and regions. Mean CFR was highest for South America at 1.973% (± 0.742) and lowest for Oceania at 0.264% (± 0.107), while the Africa sub-region recorded the lowest scores for pandemic preparedness, vaccination rate, and other indicators. Population Median Age [0.073 (0.033 0.113)], Vaccination Rate [−3.3389 (−5.570.033 −1.208)], and Inequality-Adjusted Human Development Index (IHDI) [−0.014 (−0.023 −0.004)] emerged as statistically significant predictors of COVID-19 CFR, with directions indicating increasing Population Median Age, higher inequalities in human development and low vaccination rate are predictive of higher fatalities from COVID-19.

**Conclusion:**

Regional differences in COVID-19 CFR may be influenced by underlying differences in sociodemographic and pandemic mitigation indicators. Populations with wide social inequalities, increased population Median Age and low vaccination rates are more likely to suffer higher fatalities from COVID-19.

## Introduction

First reported in late 2019, the meteoric rise to pandemic status of the Severe Acute Respiratory Syndrome Coronavirus 2 (SARS-CoV-2), commonly referred to as COVID-19 [1] has exerted untold distress on virtually every aspect of human life [2, 3]. As health scientists and practitioners scramble to deescalate the pandemic with standard public health measures including approved vaccines, COVID-19 continues its relentless rage through human populations, leaving behind a long trail of growing fatalities and overwhelmed health systems around the world [4, 5].

Whereas, there is a general fluidity of emerging information around COVID-19 as we learn more about this virus and pandemic daily [6], there appears to be some consistency in its disproportionate burdens on older individuals and those with some underlying medical conditions such as obesity, diabetes and heart disease [7, 8]. To the extent that older people appear to be more vulnerable to COVID-19, and more likely to die after contracting the disease, it is conceivable that countries with relatively younger populations may have lower case fatality rates (CFRs) compared to countries with older populations.

As more data from the pandemic becomes available, wide variations in COVID-19 case-loads and associated fatality rates within and across countries with varied socioeconomic and health metrics have incited a growing research interest in the effect of social indicators and health system capacity on COVID-19 case-loads and associated fatality rates [2, 6, 9–12]. A study published in 2020 found significant associations between COVID-19 CFRs, GDP per capita, doctor-patient ratios and health expenditure per capita, leading them to project worse COVID-19 CFRs for low-income countries [9]. In another publication, researchers projected worse COVID-19 outcomes for countries with poor health infrastructure and capacity [10]. A recent study in the US also found higher COVID-19 CFRs among poorer neighborhoods [11] while another publication in the same period attributed the low COVID-19 CFR observed in Africa to lower population mean age and related lower life expectancy resulting in reduced elderly population [12].

In this research paper, we investigated the effect of pandemic preparedness, critical care capacity, vaccination rate, population median age, and inequalities in human development on country-specific COVID-19 CFRs at the global level to allow for cross-country and regional comparisons. We then used regional analysis to highlight regional differences in COVID-19 CFRs.

## Methods

### Study variables and data collection

COVID-19 CFR was the outcome variable, which was calculated as the percentage of confirmed deaths due to COVID-19 relative to the total number of confirmed cases of COVID-19. The data used in determining the country specific COVID-19 CFRs was obtained from the World Health Organization (WHO) COVID-19 data output for January 26, 2022 [13].

In addition to the population median age by country (2021 data obtained from Worldometer) [14], four other indicators assessing pandemic preparedness (Global Health Security Index), clinical capacity for critically ill COVID-19 patients (Intensive Care Unit—ICU bed capacity), full COVID-19 vaccination rate per 100 population (Vac/100), and inequalities in human development (Inequality-Adjusted Human Development Index—IHDI) were selected as explanatory variables in the study. Country-level data for the Global Health Security Index (GHSI) were obtained from the Johns Hopkins Center for Health Security [15], Intensive Care Unit (ICU) bed capacity by country from a document developed from multiple sources [16], COVID-19 vaccination rate per 100 population (Vac/100) from the World Health Organization COVID-19 data board for January 26, 2022 [17] and data for IHDI were obtained from the United Nations Development Program (UNDP) [18].

Median age by country is an important index that summarizes the age distribution of a country by dividing the population into two numerically equal-sized groups such that, half of the people are younger than this age and half are older [19]. Since available evidence suggests a higher mortality burden from COVID-19 among older individuals [7, 8], median age by country was included in this study to evaluate the effect of age on COVID-19 CFR at the population level for the purposes of cross-country and regional analyses.

The GHSI is a premiere assessment of the capabilities of independent countries to deal with current and emerging infectious disease epidemics and pandemics such as the ongoing COVID-19 pandemic. First launched in 2019, the GHSI as a composite index incorporates several factors critical to mitigating pandemics including country specific health system strength, capacity for prevention, early detection and reporting of cases and as well as rapid response to prevent further spread [15].

A number of vaccines have been developed in response to the ongoing COVID-19 pandemic, and a growing body of research has consistently demonstrated significant reductions in severity of disease and associated mortality from COVID-19 among individuals who are fully vaccinated against the disease [20, 21]. Nevertheless, vaccination rates continue to vary widely within and across national boundaries [13] for many reasons including unequal access to vaccines within and across countries. Given the wide variations in country-specific COVID-19 vaccination rates, we included the vaccination rate per 100 population (Vac/100) to investigate the influence country-specific vaccination rates on global COVID-19 CFRs.

The IHDI was added to evaluate the effect of inequalities in human development among populations on COVID-19 CFR. The IHDI incorporates inequalities in the Human Development Index (HDI) by looking at the distribution of measures of health (based on life expectancy at birth), education (based on mean and expected years of schooling) and income (based on Gross National Income Per Capita) used in estimating HDI within a population [22]. Since current evidence suggests COVID-19 related deaths disproportionately affect poorer individuals, communities, minority and marginalized groups in the USA [23], we wanted to explore the effect of inequality in human development on COVID-19 CFRs at the global level.

As the most severe and complicated cases of COVID-19 are associated with significant risk of mortality and require specialist care that is better received from ICUs to optimize chances for survival and recovery, ICU bed capacity per 100,000 population was included in the current study with the hypothesis that country level ICU capacity will influence COVID-19 CFRs.

### Data analysis

After merging and cleaning the data, we dropped all countries that had one or more missing data points for any of the selected variables. A preliminary analysis was then used to identify outliers based on CFR value in the data that were also dropped. Out of 190 countries, 74 countries either reported zero COVID-19 cases and/or deaths, or had at least one missing explanatory variable for which reason we dropped them from the study. Of the remaining 116 countries with complete set of variables, we dropped 6 countries with CFR higher than 5 that emerged as outliers in our preliminary assessment of the data that included Ecuador (5.04), Egypt (5.44), Sudan (6.10), Mexico (6.50), Peru (6.94), and Yemen (18.85), leaving us with a final data set of 110 countries.

Descriptive statistics was then used to determine the normalcy or otherwise in the distribution of each variable as a basis for deciding the types of analyses to use. Based on the initial analyses, the individual variables yielded skewness values ranging from a high of 1.605 for ICU bed capacity per 100,000 population to a low of -0.408 for vaccination rate per 100 population while the Kurtosis values ranged from a high of 2.915 for ICU bed capacity per 100,000 population to a low of -1.312 for median age. The Skewness and Kurtosis values of all variables fell within the ranges considered acceptable for normally distributed data according to Hair et al. [24]. Further reinforced by a visual inspection of the curves on the histograms of each variable, we proceeded to use parametric tests for the main analyses in the study. First, we conducted descriptive statistics (Means and Standard Deviations) for the outcome and all explanatory variables. To determine suitability for the regression analysis, we proceeded to conduct multicollinearity diagnostics of the complete dataset, the results of which showed variance inflation factor (VIF) ranging from the high of 6.99 for IHDI to the low of 1.70 for ICU per 100,000 population. Since these VIF values fall below the acceptable recommended maximum VIF value of 10 according to Hair et al. [25], we proceeded to conduct multiple regression. analysis to determine if any of the explanatory variables predicted global COVID-19 CFRs.

## Results

### Global distribution of CFR

**Fig. 1:**
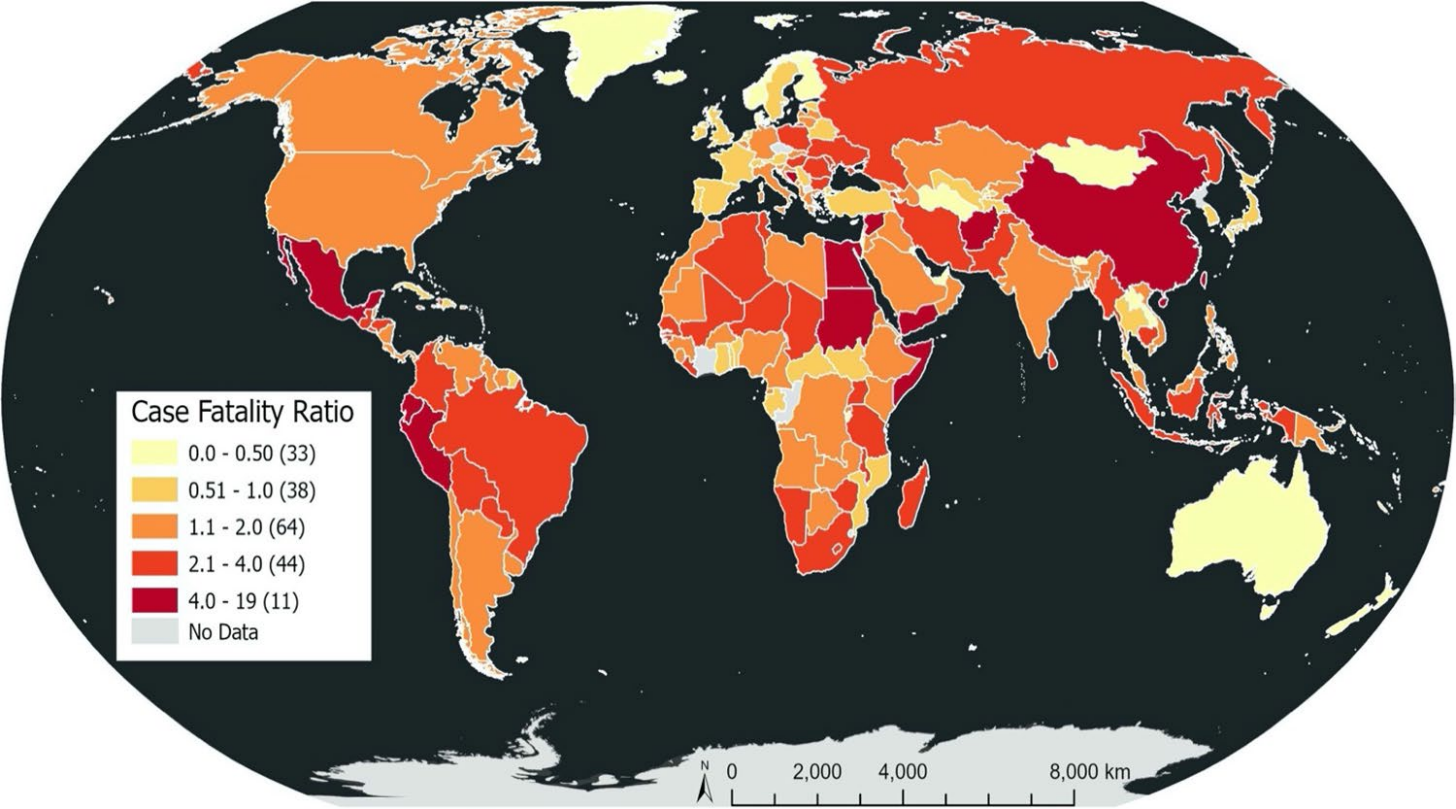
Choropleth Map of COVID-19 CFR (Case Fatality Rate) for 190 countries (The number in the parenthesis refer to the number of countries in the CFR range)

Based on the available data, 190 countries were mapped to show the distribution of CFR at global level as illustrated in Fig. 1. CFR for 33 countries ranged from 0 to 0.5%, 38 countries ranged from 0.51 to 1%, 64 countries ranged from 1.01% to 2%, and 44 countries ranged from 2.01 to 4% whereas CFR for 11 countries was higher than 4%. Greenland and Iceland recorded very low number of deaths as compared to the number of cases yielding CFRs of 0.04% and 0.07% respectively. Conversely, Yemen had exceptionally high CFR (18.85%), whereas, Mexico, Peru, and Sudan also had CFR more than 6%.

### Descriptive analysis

The descriptive analysis indicated in Tables 1 and 2 included 110 countries at the global level—26 African countries, 27 Asian countries, 32 European countries, 13 North and Central American countries, 10 South American countries and 2 Oceanian countries. The average CFR was 1.544% of the total cases recorded at the global level. Iceland recorded the minimum CFR of 0.075%, whereas, Bosnia and Herzegovina recorded the maximum CFR of 4.215%. For the regional analysis, mean CFR was highest for South America (1.973%) and lowest for Oceania (0.264%).

**Table 1:** Descriptive statistics for COVID-19 CFR and selected Socioeconomic Indicators

Variables	N	Mean	Standard deviation	Minimum	Maximum
CFR (%)	110	1.544	0.910	0.075	4.215
Median Age	110	32.03	9.087	15.8	47.3
IHDI	110	0.640	0.183	0.303	0.899
ICU	110	6.89	7.09	0.1	34.7
Vac/100	110	48.597	25.935	0.696	88.102
GHSI	110	44.657	13.519	20.9	75.9

Case Fatality Rate (CFR), Median Age, Global Health Security Index (GHSI), Inequality-adjusted Human Development Index (IHDI), Vaccination rate (Vac/100), ICU Beds Per 100,000 Population (ICU)

**Table 2:** Descriptive statistics for regional analysis of COVID-19 CFR and selected indicators

Variables	Regions	Africa [26]	Asia [27]	Europe [32]	N and C America [13]	S America [10]	Oceania [2]
CFR (%)	Mean	1.804	1.488	1.310	1.654	1.973	0.264
	SD	0.779	0.961	0.980	0.780	0.742	0.107
Median Age (years)	Mean	22.211	30.889	41.624	30.308	30.538	38.300
	SD	6.794	6.720	2.670	6.431	3.593	0.566
IHDI	Mean	0.434	0.641	0.818	0.601	0.636	0.772
	SD	0.125	0.140	0.062	0.141	0.117	0.124
ICU	Mean	1.681	6.481	11.206	6.000	9.613	6.400
	SD	2.612	6.919	6.067	9.280	7.669	3.818
Vac/100	Mean	21.782	51.099	62.600	52.204	59.332	79.395
	SD	23.295	23.011	16.594	20.127	19.734	1.247
GHSI	Mean	32.637	44.567	54.706	43.131	41.025	66.800
	SD	7.445	11.648	9.000	15.529	12.414	6.081

Standard Deviation (SD), Case Fatality Rate (CFR), Median Age, Inequality-adjusted Human Development Index (IHDI), ICU Beds Per 100,000 Population (ICU), Vaccination rate (Vac/100), Global Health Security Index (GHSI)

Median age analysis revealed a mean of approximately 32.2 years and standard deviation of approximately 9.1 years at the global level. Uganda recorded the minimum median age of 15.8 years and Japan recorded the maximum with 47.3 years. For the regional analysis, Africa recorded the minimum median age of 22.2 years while Europe recorded the maximum median age of 41.6 years.

Analysis for GHSI showed an average of 44.657 with standard deviation of 13.519 at the global level. Venezuela recorded the minimum GHSI of 20.9 and USA recorded the maximum of 75.9. For the regional analysis, Africa recorded the minimum GHSI of 32.6 while Oceania recorded the maximum GHSI of 66.8. Concerning IHDI, the global average score was 0.640 with standard deviation of 0.183. Comoros recorded the minimum IHDI of 0.303 and Norway recorded the maximum of 0.899. IHDI was also the lowest for the Africa region (0.434) and highest for Europe (0.818).

The average vaccination rate per 100 population (Vac/100) globally was 48.597 with standard deviation of 25.935. Haiti recorded the minimum vaccination rate of 0.696, while Chile recorded the maximum vaccination rate of 88.102. Regional analysis showed Mean Vac/100 was highest for Oceania (79.395) and lowest for Africa (21.782).

Global analysis showed an average ICU beds per 100,000 population as 6.89 with a standard deviation of 7.09. Malawi recorded the minimum ICU beds per 100,000 population of 0.1 and USA recorded the maximum of 34.7 ICU beds per 100,000 population. Africa recorded the minimum ICU beds/100,000 population of 1.68 while Europe recorded the maximum.

### Inferential analysis

**Fig. 2:**
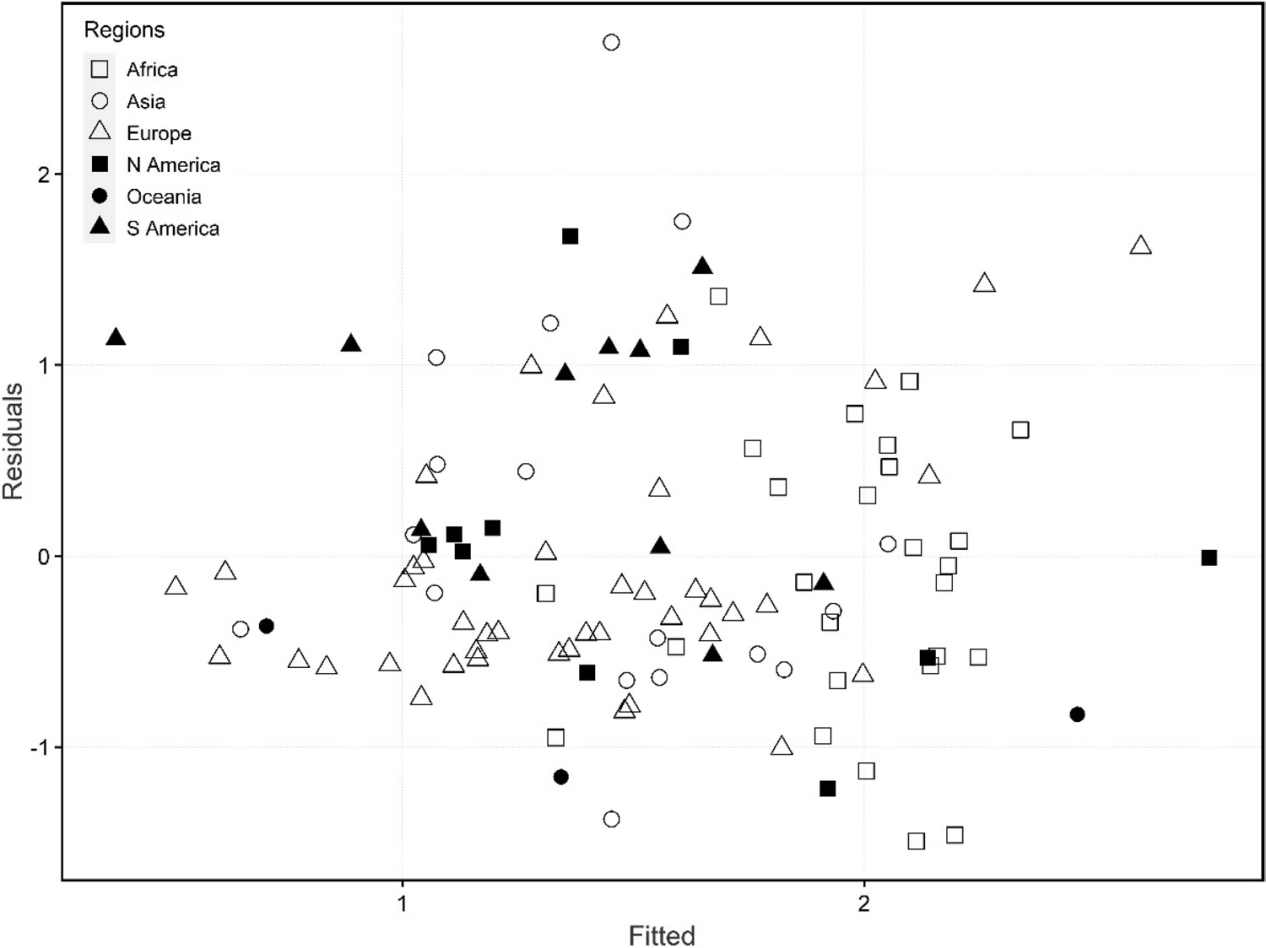
Residual plot of the regression model

Table 3 provides a summary of the inferential results on the final Ordinary Least Squares (OLS) model generated by regressing CFR on the explanatory variables. The regression model was statistically significant *F* (5, 104) = 7.907, *p* < 0.001 and *R*^2^ being 27.5%. This meant the independent variables (median age, IHDI, ICU, Vac/100 and GHSI) collectively accounted for 27.5% of the variance in COVID-19 CFR. More importantly, IHDI, country median age and Vac/100 emerged as statistically significant predictors of Global COVID-19 CFR in the final regression model with directions suggesting wider inequalities in the HDI and increasing population median age may be associated with higher COVID-19 CFR while higher vaccination rate against COVID-19 is associated with a lower burden of COVID-19 CFR. Figure 2 depicts a plot of regression residuals vs fitted CFR values with no variation across regions.

**Table 3:** Final regression model for Covid-19 Case Fatality Rate Predictors

Variables	B [95% CI]
Intercept	2.257 [1.576 2.937]
Median Age	0.073 [0.033 0.113] *
IHDI	−3.389 [−5.570 −1.208] *
ICU	0.001 [−0.026 0.029]
Vac/100	−0.014 [−0.023 −0.004]*
GHSI	−0.005 [−0.024 0.014]
F Statistic	7.907
P-Value	< 0.001
R^2^	0.275

Median Age, Inequality-adjusted Human Development Index (IHDI), ICU Beds Per 100,000 Population (ICU), Vaccination rate (Vac/100), Global Health Security Index (GHSI)

^*^Significant at 95% CI

## Discussion

A country’s pre-existing socio-economic and health service capacity may influence its overall ability to effectively respond to the pandemic and may account for differences in COVID-19 CFRs across countries and regions. Earlier studies at the outset of the COVID-19 pandemic identified and/or projected socio-economic factors such as median age, life expectancy, GDP per capita and health indicators such as current health expenditure, number of hospital beds and medical doctors to be associated with COVID-19 CFR [9, 12, 25, 26]. In this study, current COVID-19 data was used to investigate the effect of inequalities in human development, population median age, pandemic preparedness, vaccination rates, and clinical capacity on COVID-19 CFRs across countries and regions. Regional analysis was also used to highlight regional differences in COVID-19 CFRs. We were especially keen to examine what influence socioeconomic inequalities (IHDI) and vaccination rates exerted on COVID-19 CFRs across countries and regions. It is important to add that, the use of current COVID-19 data allowed us to compare the current COVID-19 case fatality burden in the context of cross-country and regional variations of the selected indicators against projections made for various regions in the early days of the COVID-19 pandemic.

Descriptive data for specific regions showed the African region reported the lowest scores for all the selected indicators included in the study. Surprisingly, Mean CFR was highest in the South American region, though the African region followed closely. Africa and South America had the lower average median ages compared to Europe and Oceania. This result may give credence to the argument that low resourced countries, particularly those in Africa and South America, may have significantly younger populations in comparison to more economically developed regions like Europe and Oceania. Similarly, Africa and Asia reported the lowest regional scores for GHSI and COVID-19 vaccination compared to Europe and North and Central America. This supports the need for innovative public health interventions for resource poor regions.

Regional variations in GHSI, IHDI, ICU bed capacity and vaccination rates may be a reflection of variations in pre-existing socio-economic and health resources, and these findings suggest that health care capacity in low resourced regions may be poor in comparison to more economically developed regions. Consequently, it is expected that these low resourced regions may be inadequately prepared to respond to the COVID-19 pandemic resulting in a higher vulnerability to fatalities from COVID-19. A close look at the choropleth map (Fig. 1) generated for all countries depicts wide variations in COVID-19 CFR across all regions of the world, without a clear and consistent pattern in any specific region, which was found to be very similar to the choropleth map generated for only countries included in the model that also had no consistent pattern in any region. Indeed, we acknowledge the heterogeneity of study variables such as population median age within regions, and the possible effects these within region differences may exert on the regional analysis cannot be ignored.

Country median age, IHDI, and vaccination rate emerged as statistically significant predictors of COVID-19 CFR from the regression analysis. Specifically, for median age, the results from the regression analysis revealed that higher country median age is predictive of higher COVID-19 CFR. This finding suggests that countries located in Europe, Oceania and North and Central America with higher rates of older populations may experience higher COVID-19 CFRs than countries with younger populations, such as those in Africa, South America, and Asia. These findings are consistent with earlier studies that found that higher COVID-19 CFR among higher age groups in multiple regions [12, 26, 27]. A possible explanation for this result is that many older adults may have pre-existing comorbidities such as diabetes, cardiovascular diseases, or hypertension [28]. Consequently, older adults with comorbidities have the highest susceptibility to COVID-19 fatality [29].

Regarding the emergence of the IHDI as a statistically significant predictor of COVID-19 CFR, the results indicate higher inequalities in human development is predictive of higher COVID-19 CFR. In other words, countries with wide inequalities in human development would have higher fatality rates from COVID-19, while countries with lower inequalities in human development would have lower fatality rates from COVID-19. To be clear on the importance of this finding, IHDI evaluates inequalities in human development by factoring in a measure of the dispersion in the distribution of the measures of health, education, and income used in estimating HDI within a population [22]. Individuals in the lower socioeconomic brackets are more likely to have limited access to preventive and treatment opportunities for COVID-19 for various reasons including financial constraints, leading to higher vulnerabilities to COVID-19. This finding is a strong reflection of the effect of underlying inequalities in socioeconomic factors, leading to a disproportionate burden of COVID-19 case fatalities on individuals and communities in the lower socioeconomic brackets and is consistent with the findings of earlier studies that showed worse COVID-19 outcomes for poorer communities [11]. This reinforces the urgency for a concerted effort of global investments to support all countries, but especially economically disadvantaged countries.

Vaccination rate emerged as the final predictor of COVID-19 CFR from the regression analysis, with a direction suggesting countries with higher vaccination rates recorded lower overall mortality from COVID-19. This finding is consistent with extant public health recommendations on the efficacy of COVID-19 vaccinations in significantly reducing the risk and associated burden of severe disease, hospitalization, and mortality from COVID-19 [20, 21]. It is essential to add this finding also underscores the importance of COVID-19 vaccines in the crusade against the pandemic on the global stage. This is especially important in the face of existing inequalities in the distribution and access to COVID-19 vaccines across countries, a situation if allowed to continue, may inevitably lead to the emergence of new COVID-19 variants that may further prolong the pandemic [30] along with the many disruptions to human life that come with it. Concerted efforts among countries and regions in the global community including equitable distribution of resources, such as COVID-19 vaccines, will therefore be critical to the success of the fight against the pandemic.

To the extent that past researchers reported mixed results of varied COVID-19 outcomes between rural and urban populations at different times during the pandemic [31, 32], we tested the influence of urbanization on the COVID-19 case fatality rates in the context of the predictor variables. However, including a measure of urbanization in the regression model did not meaningfully influence the results produced which were similar to the analysis without the measure of urbanization with the same predictor variables—country median age, IHDI, and vaccination rate again emerging statistically significant predictors of COVID-19 CFR. The measure of urbanization was therefore not included in the final result.

This study is not without its limitations. First, many countries differ in their definition of COVID-19 related deaths. Subsequently, there may be much variability in recording COVID-19 CFR between countries [33]. Additionally, this variability in recording may also be attributed to the distribution of resources in low resource countries compared to high resource countries. High resource countries may implement more strategies for correctly classifying whether or not a death was COVID-19 related [27]. Conversely, low resourced countries may have missed deaths attributable to COVID-19 resulting from lack of testing and surveillance [34]. Both of these factors may contribute to an overestimation or underestimation of COVID-19 CFR respectively. There is also additional difficulty in assessing COVID-19 CFR rates because of the dynamic nature of the COVID-19 pandemic in the face of variations in the burden of disease due to country-specific and regional differences in dominant variants at different points in time, as well as variations in public health measures, priorities and responses as influenced by other factors unique to each country and/or region. Finally, the lack of data on some of the variables of interest across some of the countries is considered a limitation in this study. These limitations notwithstanding, we consider the use of current COVID-19 data from the World Health Organization, inclusion of only countries with data on all the variables of interest as well as the use of regional analysis, to highlight regional differences in COVID-19 CFR as strengths in this study.

## Conclusion

Lower Country Median Age may have played a significant role in mitigating the projected higher COVID-19 CFRs among less-resourced countries in regions such as Africa in the early phases of the pandemic. Nevertheless, the emergence of low vaccination rate and wide inequalities in human development as significant predictors of COVID-19 CFR still calls for proactive, collaborative efforts toward narrowing and bridging the gaps in the distribution of socioeconomic resources as well as vaccines against COVID-19 among especially less-resourced countries to optimize responses to the ongoing pandemic and prepare them for the next one which benefits the global community overall. Finally, given the flux situation of the pandemic, further research is recommended to deepen our understanding of the various factors associated with morbidity and mortality from COVID-19.

## Data Availability

We used publicly available secondary data and will readily share the specific data set used upon request.

## Code availability

We used SPSS and ArcGIS for the data analysis.
